# 

**Published:** 1905-09

**Authors:** 


Homœopathy to the Rescue.

VOL. XXI. SEPTEMBER, 1905. No. 3.

### TEXAS

### Medical Journal.

TWENTY-FIRST YEAR.

LIBRARY
SURGEON GENERAL'S OFFICE
SEP. 14 1905

SUBSCRIPTION, $1.00 A YEAR, IN ADVANCE.

SINGLE COPIES, 10 CENTS.

PUBLISHED AT AUSTIN, TEXAS, BY

F. E. DANIEL, M. D.,

PROPRIETOR.

PRINTED BY THE VON BOECKMANN-JONES CO.

Entered at the Postoffice at Austin, Texas, as Second-class Mail Matter.

